# A narrative review of adaptive testing and its application to medical education

**DOI:** 10.12688/mep.19844.1

**Published:** 2023-10-24

**Authors:** Steven A. Burr, Thomas Gale, Jolanta Kisielewska, Paul Millin, José M. Pêgo, Gergo Pinter, Iain M. Robinson, Daniel Zahra

**Affiliations:** 1 University of Plymouth, Plymouth, England, UK; 2 University of Minho, Braga, Portugal; 3 Lancaster University, Lancaster, England, UK

**Keywords:** Assessment, adaptive testing, personalised, progress testing, fairness, different questions, reliability, compensation

## Abstract

Adaptive testing has a long but largely unrecognized history. The advent of computer-based testing has created new opportunities to incorporate adaptive testing into conventional programmes of study. Relatively recently software has been developed that can automate the delivery of summative assessments that adapt by difficulty or content. Both types of adaptive testing require a large item bank that has been suitably quality assured.

Adaptive testing by difficulty enables more reliable evaluation of individual candidate performance, although at the expense of transparency in decision making, and requiring unidirectional navigation. Adaptive testing by content enables reduction in compensation and targeted individual support to enable assurance of performance in all the required outcomes, although at the expense of discovery learning.

With both types of adaptive testing, candidates are presented a different set of items to each other, and there is the potential for that to be perceived as unfair. However, when candidates of different abilities receive the same items, they may receive too many they can answer with ease, or too many that are too difficult to answer. Both situations may be considered unfair as neither provides the opportunity to demonstrate what they know. Adapting by difficulty addresses this. Similarly, when everyone is presented with the same items, but answer different items incorrectly, not providing individualized support and opportunity to demonstrate performance in all the required outcomes by revisiting content previously answered incorrectly could also be considered unfair; a point addressed when adapting by content.

We review the educational rationale behind the evolution of adaptive testing and consider its inherent strengths and limitations. We explore the continuous pursuit of improvement of examination methodology and how software can facilitate personalized assessment. We highlight how this can serve as a catalyst for learning and refinement of curricula; fostering engagement of learner and educator alike.

## Introduction

Tests that adapt by difficulty have historically been referred to as computerized adaptive testing, or CAT ( Collares & Cecilio‐Fernandes, 2019; Gershon, 2005; Linden *et al*., 2000; Meijer & Nering, 1999; Wainer *et al*., 2000). Test items representing a range of difficulties are pre-calibrated ( Rice *et al*., 2022). During delivery, the adaptive algorithm aims to select items with a 50/50 chance of candidates responding correctly. If a candidate answers an item incorrectly then the next item they receive is easier; and if they answer correctly then the next item is more difficult. The difficulty of items thus fluctuates to find the level of ability of the candidate, at which point they will be answering half of the items they receive correctly and half incorrectly. It is implicit that candidates will each receive different items, and all expect to reach a 50% probability of getting the correct answer and thereby achieve an overall score approaching 50%. Decisions about the adequacy of performance by individual candidates are then based on the level of difficulty of the items they have gravitated toward. With most items pivoting around the level of ability of the individual candidate, it follows that the measure of their achievement is more reliable ( Lord, 1980). The main advantages of CAT are, therefore, increased reliability and more precise measurements ( Lord, 1980); with fewer test items needed to estimate the ability of an individual candidate. This improves efficiency but also decreases test item exposure which is a relevant factor in maintaining the security and stability of the test item bank underlying this technology. Additionally, since each candidate receives an individualized test, test delivery can be asynchronous for candidates. Thus, candidates from the same cohort can be assigned to different time slots. This can be useful when cohorts are too large for available venues as physical space and computers can be reused. The main criticism that is raises relates to the potential unfairness of the test administration, since candidates are presented with different subsets of items that may bias the sampling of content and stimulus difficulties ( Denny *et al*., 2019). To mitigate this, large banks of calibrated items need to be set up, to ensure a diverse range of content and difficulties that match a predetermined blueprint. This increases the workload, time demand, and cost to develop and maintain such item banks. Another criticism of CAT by difficulty is that it not only requires large banks of pre-calibrated items, but the item response theory models on which difficulty categories are based require large numbers of candidates in order to be robust (> 200 for even the simplest models). Thus, the successful examples cited have usually been national exams with many thousands of candidates ( Sands *et al*., 1997).

Tests that adapt by content have been developed more recently and are referred to as content adaptive progress tests or CAPT ( Burr *et al*., 2022), and are especially applicable to longitudinal approaches to knowledge testing such as the progress test format that is more commonly used in the education of health professionals. When adapting by content the knowledge required is retested in subsequent tests until every candidate has successfully demonstrated knowledge in all required content areas. If a candidate answers an item on particular content incorrectly then that content is more likely to be repeated (with a different item); and if they answer correctly then that content is less likely to be repeated. This effectively functions as a progress test ( Schuwirth & Van der Vleuten, 2012) since the growth of each candidate toward demonstrating knowledge in every required content area covered by the learning objectives of a programme can be monitored and supported over time. 

Here we explore the history of adaptive testing, its evolution over time, and potential implications for using different types of adaptive testing in the future.

## History of adaptive testing

The roots of adaptive testing can be traced back to the work of Binet & Simon (1905). Binet developed an IQ test that used a bank of items asked by a psychologist. The psychologist would use their estimate of the candidate’s ability to determine which items to start with, followed by branching to select which set of items they would ask next depending on the answers to the previous set. There was a pre-determined ceiling level of performance that would terminate the test, and the final score would be weighted by the chronological age of the candidate. These principles have continued to be utilized in IQ testing, including in the progression and termination rules of the widely used WAIS-IV ( Wechsler, 2008). During WWII sequential testing was developed, as a subcategory of assessment that specifically ended data collection once a predetermined threshold had been achieved ( Taylor & Russell, 1939; Wald, 1945), and this was then used in medical research ( Armitage, 1950). However, the methods were impractical for large-scale testing due to the time and resources required.

It was not until the advent of sufficient computing power in the 1960s that adaptive testing became a practical method for large-scale testing. In 1968, Frederick Lord developed a set of rules that could form the basis of a computerized adaptive testing algorithm for the Educational Testing Service to administer the Graduate Record Examination in the USA ( Lord, 1968). Subsequently, in the 1970s and 1980s, researchers began to develop new algorithms and models for adaptive testing. Sequential testing was intensively studied and applied to the assessment of candidates where candidates answered items one at a time until a predetermined level of performance was achieved (summarized by Weitzman, 1982). Item Response Theory (IRT) as a distinct area of study with practical applications was developed ( Lord, 1980), which provided a framework for modeling the relationship between a candidate's ability and their performance on individual test items. The IRT model allowed for more accurate and efficient adaptive testing, as it could estimate the candidate's ability level with greater precision than previous methods ( Weiss, 1985).

In the 1990s, the increasing accessibility of the internet and networked computing enabled the coordinated delivery of different items to different candidates in large cohorts. Researchers began to explore the use of adaptive testing in online learning and assessment. The Graduate Record Examination in the USA was the first large-scale high-stakes assessment to use adaptive testing ( Sands *et al*., 1997). However, by the turn of the century, adaptive testing had not become as commonplace as some had speculated ( Linacre, 2000). Take-up for summative use more widely across the education sector was slow, probably due to the need for a combination of: (1) commercial software ( Sahin *et al*., 2018), (2) computer venues, (3) academics willing to innovate, and (4) enlightened regulatory policymakers. Adaptive testing has been recognized to provide personalized learning experiences, as it can adapt the difficulty of items to the individual needs of each candidate. However, for a long time, it has been perceived to be unattainably complex to deliver summatively in comparison to established non-adapting methods of assessment. Perceptions of adaptive testing are also colored by the persistent cultural belief that for a test to be ‘fair’ everyone should receive the same items. In addition, there is the difficulty of making high-stakes decisions based on candidates answering different items. The expansion in use of adaptive testing has thus predominantly been through developing formative assessments, and this has evolved with the advent of mobile devices to support flexible personalized learning ( Choi & McClenen, 2020; Conejo *et al*., 2004; Huang *et al*., 2009; Oppl *et al*., 2017; Triantafillou *et al*., 2008). Other applications based on the principles of adaptive testing have been developed to help in the evaluation of patients, for example in aspects of mental health ( Gibbons *et al*., 2008) and arthritis ( Fries *et al*., 2009). Another success, currently familiar to many is the popular “Duolingo” mobile application that requires successful content completion before the user can “level up” using adaptive release ( Teske, 2017). There have also been relatively recent developments to deliver national assessments of proficiency in literacy and numeracy for school children using adaptive testing in Australia ( Thompson, 2017) and Wales ( Williams, 2017).

To date, the emphasis on adaptive testing has been to adapt by difficulty. Recently in 2018–22 the European Board of Medical Assessors coordinated the development of an online international progress test, that adapted by difficulty, involving 8 medical schools as partners across 5 countries. This revealed that the creation and delivery of sequential formative tests was feasible and achievable ( Rice *et al*., 2022). Other medical schools have also begun to develop tests that adapt by difficulty ( Koşan *et al*., 2019). The development of a progress test that adapts by difficulty provided the foundations to enable two of the original partner medical schools (at the universities of Plymouth and Minho) to then develop the means to adapt tests by content instead of difficulty, and to begin to practically implement this summatively ( Burr *et al*., 2022). This form of summative assessment is not only personalized to ensure performance in all required content is achieved, but also motivates, supports, and empowers candidate development, and thus functions *as* learning ( Bennett, 2010).

## Advantages of adaptive testing

One of the key advantages of adaptive testing is its ability to provide a more accurate assessment of a candidate's knowledge and skills compared to other assessment methods. In a study by Wang and colleagues, adaptive testing was shown to have higher validity, in addition to reliability, compared to traditional testing methods ( Wang *et al*., 2012). The authors concluded that adaptive testing could provide a more accurate and fair assessment of a candidate's abilities. In another study, the use of an adaptive testing method significantly reduced the testing time while maintaining the same level of accuracy as traditional testing methods ( Weiss & Kingsbury, 1984). Thus, adaptive testing can provide a more efficient testing experience, which could be improved further by considering the use of alternative statistical models to assess candidate knowledge ( Boyd *et al*., 2010). Another advantage of adaptive testing is its ability to provide personalized feedback. In a study by Martin and Lazendic (2018), candidates were assessed using an adaptive testing method that provided personalized feedback based on their performance. The results showed that candidates who received personalized feedback had higher levels of motivation and engagement compared to those who received generic feedback. It has also been suggested that adaptive testing improves the candidate’s motivation and engagement by presenting items that are challenging but not too difficult, although benefits will not accrue without curriculum alignment ( Griff & Matter, 2013). Furthermore, adaptive testing can provide valuable feedback to both the student and the educator by identifying areas of strength and weakness and guiding the learning process ( Martin & Lazendic, 2018). In contrast to tests that adapt by difficulty, tests that adapt by content are intrinsically aligned to the principles and advantages of programmatic assessment ( van der Vleuten *et al*., 2012), offering an alternative to portfolio-based assessment, with linkage between tests. A programmatic approach to assessment decreases the use of high stakes assessments for making progression decisions and instead uses continuous assessment methods which build up a profile of a learner’s growth in knowledge and skills.

## Potential limitations of adaptive testing

One of the main limitations is the potential for item exposure, where candidates may share or memorize the items and compromise the validity of the assessment ( Persky & Fuller, 2021). To minimize this risk, CAT requires a large pool of items that can be randomly selected and rotated across candidates. Nering and Ostini (2011) have previously noted that the development of large item banks can be time-consuming and costly. However, the advent of automatic item generation ( Lai *et al*., 2009) and generative Artificial Intelligence ( Falcão *et al*., 2022; Falcão *et al.*, 2023; Sun & Hoelscher, 2023) has the potential to dramatically augment the process of creating large numbers of items. Item security could be further improved if the same item could be rewritten in different ways using generative Artificial Intelligence.

An additional requirement for tests that adapt by difficulty is the need for sound psychometric models and algorithms to facilitate decisions about the setting of performance thresholds ( van der Linden & Hambleton, 2013). For example, the application of IRT and Rasch modelling to make pass-fail decisions ( Kuravsky *et al*., 2017; van der Linden & Pashley, 2009) could be considered difficult to understand and as a result, for decisions about candidates to lack transparency. Adapting by difficulty requires the selection of items ‘on the fly’ within a test, and this also means that candidates cannot be permitted to go back and change their answers to items previously viewed. Thus, navigation must be unidirectional if adapting by difficulty within a test (whereas navigation can be bidirectional if adaptation is by content between tests). When adapting by difficulty, if a candidate receives an easier item, then this indicates they have answered the previous item incorrectly. With bidirectional navigation candidates could spend time during an examination trying to evaluate the relative difficulty of sequential items and thus whether they should change their answers. The potential for differential cueing effects on easy and hard items ( Schuwirth *et al*., 1996) would become more complex and undesirable. In contrast, when adapting by content the items can be preselected before the start of each test, and bidirectional navigation retained, because adaptation occurs between tests rather than within each test.

An additional requirement for tests that adapt by content is the need for sequential test opportunities with intervals between them to give the opportunity for weaknesses to be addressed before re-testing ( Ricketts & Bligh, 2011). The potential to benefit from such feedback mandates that an indication of the content (although not necessarily the items) which could be asked is transparently known by candidates in advance; a fact that can undermine the long-established benefits of discovery learning ( Bruner, 1960; Castronova, 2002). When adapting by content, all items also need to have equal value to each other, when in practice there may be no such consensus. Furthermore, there needs to be an acceptance that the different aspects of content being tested can be acquired in any order over the series of tests, and so the assessment of some content may be out of alignment with its teaching for some candidates. However, this can be considered beneficial where there are multiple teaching sites or multiple classes within the same year, or where different placement rotations are experienced, as tests which adapt by content effectively adjust.

In comparison, a test that adapts by both difficulty and content would need to prioritize one over the other, and whichever isn’t prioritized would suffer a degree of compromise. Practically, a test cannot contain the same range of difficulty in items for the same range of content, for all candidates, and still adapt by both simultaneously; a trade-off is required ( Luecht *et al*., 1998). If prioritizing difficulty, then candidates will need to receive items covering different aspects of content in order to balance the overall difficulty of the test. Similarly, if prioritizing content, a test cannot cover as wide a range of difficulty and provide assurance all content is achieved to the same standard. To completely adapt by both difficulty and content, all content areas would need to be covered at each difficulty increment, or vice versa, requiring additional items, such that it becomes impractical to vary both factors within a restricted test length. The balancing of content within tests which adapt by difficulty is a well-known issue with several approaches available to control it ( Leung *et al*., 2003; Veldkamp & van der Linden, 2002). An equivalent combination of adapting by difficulty whilst simultaneously evaluating cognitive attributes has also been developed ( Gierl & Zhou, 2008), although the number of domains is limited and decision making more complex.

## Comparison with other assessment methods

Adaptive testing is a method of assessment where each item presented to a candidate is based on their previous answers. Adaptive testing is important for developing personalized assessments: (1) to gain more reliable measures of individual performance, (2) to ensure acquisition of all the required learning outcomes by all candidates, and (3) to tailor individual support for learning. Tests can be designed to adapt by item difficulty, content, or both. 

Except for some specific circumstances such as test-equating, other methods of assessment typically present all candidates with the same items: (1) When considering difficulty this means that high ability candidates will receive many items that they find easy, while other low ability candidates will receive many items that they find hard. A lower proportion of items are close to the level of ability of any given candidate and so the reliability of the measures of performance will be lower than if the test adapts. (2) When considering content this means that, unless the required performance threshold is 100%, there will be a degree of compensation whereby some of the content will not need to be demonstrated. Candidates can thus avoid certain content areas and still achieve a pass. When considering these points, adaptive testing should work more efficiently to practically facilitate learning by providing support in accord with the theory of the zone of proximal development ( Vygotsky & Cole, 1978).

## Why it is fairer to ask candidates different questions

What is fairness? Is it that candidates are all treated the same? Is that possible, or reasonable? Is it fairer for candidates to be asked the same questions or different questions? At face value, it may appear fairer for everyone to be judged by their answers to the same set of questions. However, a moment’s reflection might call to mind complexities such as those parodied by the Traxler cartoon of a monkey, elephant, goldfish, and other animals being assessed ‘fairly’ by all being asked to climb a tree ( Traxler, 1983). This equality approach to assessment presupposes all candidates have the same preparatory experience, as well as being matched in all other variables. If candidates cover the content of the curriculum at different times through different learning experiences, but are given the same set of questions at the same time with which to achieve a particular pass mark, then the unfairness in this equality approach should be apparent. In such circumstances, it becomes fairer to ask candidates different questions to each other.

The idea of asking candidates different questions to each other might be countered by claiming that candidates receive the same learning and teaching within a programme. However, it is typical of most contemporary curricula in higher education ( Clegg *et al*., 2010; although admittedly less so in school education; McNeil, 2014), for candidates to have different amounts of time on different content. This can occur: in small group work, with different peers; by choosing different coursework assignment titles; by selecting or been limited to different placement opportunities; and due to a greater emphasis on self-directed learning. Thus, undergraduate candidates may cover content in different orders, and to different extents (or not at all), compared to other candidates in the same cohort. This variation in experience is compounded by differences in attendance through illness and other valid extenuating circumstances, leading to alternative replacement coursework, resit examinations, or even the repeating of parts of a programme with a different cohort ( Burr *et al*., 2018). Thus, traditional assessment strategies already allow that candidates on the same programme will be graduated (or not) based on a different set of assessments and assessment items to each other. It is therefore not currently the case, nor a reasonable expectation of new assessment designs, that candidates all be judged only by their response to the same questions during a programme of study. With this in mind, programmes are often designed with the end-point being for candidates to demonstrate their achievement in covering a set of learning objectives.

The traditional model of assessment presents all candidates with the same set of items covering all learning objectives, and requires them to achieve a particular pass mark with those items ( Holzinger *et al*., 2020). This has as its graduation criteria the achievement of a particular pass mark across assessments, rather than the demonstration of all the learning objectives. Thus, candidates can compensate for missing some learning objectives by excelling in others ( Brinkman *et al*., 2017), unless all assessments have a 100% pass mark ( Herold *et al*., 2016). This can even be to the point of completely ignoring one or more objectives or content areas. Furthermore, achievement of the pass mark is based on the same items set for all candidates. Within a curriculum where learning experiences differ, this means that not all candidates are given the same opportunities to develop and demonstrate their knowledge and meet the pass mark. Unmet objectives are never revisited if the pass mark has been achieved, and as such, the end-point for candidates is different ( Bierer *et al*., 2008). Some candidates will graduate with a broad range of knowledge, whereas others will graduate with exceptional knowledge of some areas and less (or no) knowledge of others ( Cummings *et al*., 2019).

In contrast, requiring candidates to demonstrate achievement of knowledge in each content area by the end of the programme is unquestionably more aligned to the stated aim of creating graduates that have demonstrated achievement of *all* of the learning objectives. Unlike the previous situation, in this case, all students have the same end-point of demonstrating achievement of each and every learning objective, and are given repeated opportunities to demonstrate this through assessment items that adapt by content. The selection of items is personalised to each candidate, with tailored feedback and support to meet objectives that remain unmet after each assessment. This adaptation, to ensure the same end-point is met by each graduate, necessitates each candidate receiving different assessment items over the course of the programme. Candidates have been shown to perceive alternative assessment types as fairer where there is the opportunity to react to feedback and stimulate deeper learning ( Struyven *et al*., 2005). In addition, the approach fosters self-directed learning, a form of individualised learning which is realisable with asynchronous delivery ( Svedberg, 2010). Individualised learning can then also be assessed, with the common end-point of meeting all objectives, through adapting items ( Burr *et al*., 2022).

Considerations of fairness also tend to focus on the potential for cheating, which can be ameliorated by presenting different questions to candidates in the same cohort ( Denny *et al*., 2019). It has long been established that fairness also depends on candidate perceptions that assessments are authentic, reasonable, realistic, developmental, and beneficial ( Sambell *et al*., 1997). It is clear that fairness is a multidimensional construct, perceived through many different lenses. Contrary to the recurrent, yet impractical, position that fairness depends on everyone facing the same questions, it can in fact be fairer, as explained here, to ask candidates different questions. This is particularly true where repeated personalised assessments are possible, giving a fairer opportunity for all candidates to demonstrate achievement of all the learning objectives, and to ensure the same standard is achieved.

## Conclusions

Whilst the ideas underpinning adaptive testing have been around since the early 1900’s, examples of its use are scarce until the later part of that century, when the widespread availability and use of computers made it possible to implement adaptive assessment methods on a large scale. Today, the recent development of software to comprehensively deliver tests that adapt by difficulty and content facilitates easier adoption of more personalized forms of summative assessment, and with it, the advantages of increased assessment reliability, and the assurance that each candidate is meeting all the required learning outcomes, with tailored individual support for learning and feedback where it is needed. These technical developments to facilitate the delivery of adaptive testing do not necessarily overcome other barriers such as the misguided perception that it is unfair for candidates to be required to answer different questions. However, in cases where assessment across a wide range of content is paramount, the use of different questions could become accepted if there are significant benefits. This could certainly be the case for adapting testing by content since this can eliminate compensation and thereby categorically demonstrate attainment in all of the knowledge required.

## Data Availability

There are no data associated with this article.
