# Supplementary material for: A narrative review of adaptive testing and its application to medical education
Source: MedEdPublish (2016). 2023 Oct 24;13:221. [Version 1] doi: 10.12688/mep.19844.1 (PMC10680016; doi:10.12688/mep.19844.1)
Supplement: Peer review history

# Reviewer report: Reviewer response for version 1

**DOI:** 10.21956/mep.21260.r35254

**Published:** 2023-12-22

**Authors:** S Helen Oram

**Affiliations:** 1 King's College London, London, UK

The narrative review by Burr et al. delves into the intriguing realm of adaptive testing and its application in medical education. The authors meticulously trace the historical evolution of adaptive testing, shedding light on its lesser-known but significant history. The advent of computer-based testing, as discussed, has not only facilitated the integration of adaptive testing into conventional study programs but has also spurred the development of software capable of automating summative assessments that adapt by difficulty or content.

 The distinction between adaptive testing by difficulty and content is eloquently presented. Adaptive testing by difficulty, often referred to as computerized adaptive testing (CAT), involves selecting items based on a candidate's performance. If an item is answered correctly, the subsequent one is more challenging, and vice versa. While this method enhances reliability and precision, concerns about fairness arise as candidates are presented with different subsets of items. On the other hand, adaptive testing by content, exemplified by Content Adaptive Progress Tests (CAPT), focuses on retesting knowledge until every candidate demonstrates proficiency in all required content areas. This approach ensures assurance of performance in all outcomes but may sacrifice some aspects of discovery learning.

 The review thoroughly explores the educational rationale behind the evolution of adaptive testing, considering its strengths and limitations. The continuous quest for improving examination methodology, coupled with the role of software in facilitating personalized assessment, is eloquently discussed. The authors emphasize how adaptive testing can act as a catalyst for refining curricula, fostering engagement among learners and educators alike.

 A significant portion of the review is dedicated to the historical progression of adaptive testing, tracing its roots back to Binet and Simon's work in 1905. The subsequent development of computerized adaptive testing in the 1960s, driven by increased computing power, laid the foundation for the more recent advancements discussed in the paper. The slow adoption of adaptive testing for summative purposes in education is attributed to a combination of factors, including the need for commercial software, suitable venues, innovative academics, and supportive regulatory policies.

 The advantages of adaptive testing, both by difficulty and content, are thoughtfully presented. CAT, with its ability to provide individualized tests asynchronously, offers increased reliability and efficiency, albeit with the challenge of potential unfairness due to varying item subsets. Content adaptive progress tests, on the other hand, provide targeted support and motivation for candidates to demonstrate knowledge in all required content areas, addressing some of the limitations of adaptive testing by difficulty.

 The authors astutely discuss potential limitations of adaptive testing, including item exposure concerns and the need for large item banks. They recognize the role of technology, such as generative Artificial Intelligence, in addressing these challenges, showcasing a forward-looking perspective. The manuscript also delves into the psychometric models required for tests adapting by difficulty and the necessity for sequential test opportunities with intervals for tests adapting by content.

 A distinctive aspect of the review is its exploration of the fairness of asking candidates different questions. The authors challenge the conventional belief that fairness equates to everyone facing the same questions, suggesting that candidates' varied learning experiences make this approach impractical. They argue that requiring candidates to demonstrate achievement in each content area, even if through different questions, aligns more closely with the aim of creating graduates who have met all learning objectives.

 In conclusion, the manuscript offers a comprehensive exploration of adaptive testing, weaving together historical perspectives, methodological considerations, and a thought-provoking discussion on fairness. The authors provide a well-reasoned argument for the adoption of adaptive testing, backed by examples from medical education. The inclusion of practical examples, figures, or tables could enhance visual representation and further engage readers.

 Overall, this review makes a significant contribution to the discourse on adaptive testing in medical education and invites further exploration of its nuanced facets.

Is the review written in accessible language?

Yes

Are all factual statements correct and adequately supported by citations?

Yes

Are the conclusions drawn appropriate in the context of the current research literature?

Yes

Is the topic of the review discussed comprehensively in the context of the current literature?

Yes

Reviewer Expertise:

Haematology; Medical Education; Assessments; Generative AI; Computer adaptive testing.

I confirm that I have read this submission and believe that I have an appropriate level of expertise to confirm that it is of an acceptable scientific standard.

# Reviewer report: Reviewer response for version 1

**DOI:** 10.21956/mep.21260.r35252

**Published:** 2023-11-24

**Authors:** Steven Capey

**Affiliations:** 1 Swansea University, Swansea, Wales, UK

This review provides a detailed overview of the development and potential uses of adaptive testing. The authors consider two contrasting approaches to the delivery of computerised adaptive testing (CAT). The narrative is clear and covers the beginning of adaptive testing through to the advent of CAT in education and medical education. The advantages and disadvantages of CAT are clearly discussed and appropriate solutions to many of these challenges are offered by the authors. This review goes further than describing the methodology of CAT by considering some of the philosophical issues associated with testing, fairness, validity and reliability. The benefit of these approaches to testing in medical education and programmatic approaches to assessment. Clear conclusions are drawn from the literature that will help to shape opinions on this rapidly developing assessment methodology.

Is the review written in accessible language?

Yes

Are all factual statements correct and adequately supported by citations?

Yes

Are the conclusions drawn appropriate in the context of the current research literature?

Yes

Is the topic of the review discussed comprehensively in the context of the current literature?

Yes

Reviewer Expertise:

Assessment in Medical Education

I confirm that I have read this submission and believe that I have an appropriate level of expertise to confirm that it is of an acceptable scientific standard.

# Reviewer report: Reviewer response for version 1

**DOI:** 10.21956/mep.21260.r35249

**Published:** 2023-11-23

**Authors:** Joanne Somerville

**Affiliations:** 1 The University of Western Australia, Perth, Western Australia, Australia

The narrative review describes the evolution of, and the educational application of the concept of adaptive testing in medical education, to tailor assessments to the individual learner's ability. This enhances the learner's experience and provides targeted feedback. The review discusses the strengths and weaknesses of the concept of adaptive testing and compares adaptive testing by difficulty and content. The introduction to the review is clear and comprehensive. The goals of the review and rationale for the review are clear and supported. The strengths and limitations of adaptative testing are comprehensively covered, analysed and supported with appropriate literature. Seminal studies are included to support the historical aspects of the review and literature from international experts in the field of programmatic assessment, progress and adaptive testing is appropriately cited, which verifies the discussion. The review is logically structured and clearly flows from the introduction to the discussion, application and conclusion. The authors take a non-biased view to the discussion, clearly presenting the strengths and limitations of adaptive testing and its iterations over time and to current developments.

 The authors provide a balanced and unbiased review of the different types of adaptive testing drawing appropriate conclusions supported by relevant and current literature citations. The review utilises sources to support the author's discussion but fails to critically evaluate them, however, many of the citations are seminal works or undisputed peer-reviewed research in the field. Therefore a critical analysis is not necessarily necessary. The review synthesises the supporting literature to provide novel insights into the topic. An example of this, is when the review presents a discussion around the concept of fairness.

 The main author has published in peer-reviewed journals on the topic of programmatic assessment and adaptive testing and therefore has demonstrated expertise in this area that gives credence to the depth and quality of the review. Further, the author remains objective in their synthesis of the appropriate use, adaptation and applicability of adaptive testing to current clinical assessment methods. The style and clarity of the review remain consistent in both language and style. References and citations are complete and all relevant works are cited. The conclusion accurately and appropriately summarises the key findings of the review and conflicts of interest are acknowledged.

 The review proposes a new and innovative and current use of adaptive testing to enhance student learning, promote a continuous longitudinal assessment method, which is reliable, valid and individualised to the learner's ability. Therefore the review significantly adds value to the existing literature.

 In my opinion, the review is suitable for indexing.

Is the review written in accessible language?

Yes

Are all factual statements correct and adequately supported by citations?

Yes

Are the conclusions drawn appropriate in the context of the current research literature?

Yes

Is the topic of the review discussed comprehensively in the context of the current literature?

Yes

Reviewer Expertise:

Medical education, assessment, professionlism, simulation.

I confirm that I have read this submission and believe that I have an appropriate level of expertise to confirm that it is of an acceptable scientific standard.

# Reviewer report: Reviewer response for version 1

**DOI:** 10.21956/mep.21260.r35247

**Published:** 2023-11-23

**Authors:** Kathy Cullen

**Affiliations:** 1 School of Medicine, Dentistry and Biomedical Sciences, Queen's University Belfast, Belfast, Northern Ireland, UK

This is a clearly written narrative review of adaptive testing. This is an area which is underrecognized and this review will be of interest to both undergraduate and postgraduate medical educators. It provides a succinct summary of the background and theory around adaptive testing and possible future applications. It outlines both advantages and disadvantages of this style of assessment.

Is the review written in accessible language?

Yes

Are all factual statements correct and adequately supported by citations?

Yes

Are the conclusions drawn appropriate in the context of the current research literature?

Yes

Is the topic of the review discussed comprehensively in the context of the current literature?

Yes

Reviewer Expertise:

Medical education - assessment

I confirm that I have read this submission and believe that I have an appropriate level of expertise to confirm that it is of an acceptable scientific standard.
